# Reproducibility and validity of a food frequency questionnaire among pregnant women in a Mediterranean area

**DOI:** 10.1186/1475-2891-12-26

**Published:** 2013-02-19

**Authors:** Jesús Vioque, Eva-María Navarrete-Muñoz, Daniel Gimenez-Monzó, Manuela García-de-la-Hera, Fernando Granado, Ian S Young, Rosa Ramón, Ferran Ballester, Mario Murcia, Marisa Rebagliato, Carmen Iñiguez

**Affiliations:** 1 Departamento de Salud Pública, Universidad Miguel Hernández, Ctra. Nacional 332 s/n 03550, Campus San Juan de Alicante, Spain; 2 CIBER en Epidemiología y Salud Pública (CIBERESP), Barcelona, Spain; 3 Unidad de Vitaminas. Servicio de Bioquímica Clínica Hospital Universitario Puerta de Hierro-Majadahonda Edificio Laboratorios (Peine 7), Planta 1ª c/Maestro Rodrigo, 2 28222, Madrid, Spain; 4 Centre for Public Health, School of Medicine, Dentistry and Biomedical Sciences, Queen’s University Belfast, Belfast, UK; 5 Dirección General de Salud Pública, Consejería de Sanidad, Avda Catalunya 21, 46020, Valencia, Spain; 6 Centro Superior de Investigación en Salud Pública (CSISP), Consejería de Sanidad, Avda Catalunya 21, 46020, Valencia, Spain; 7 University of Valencia, Valencia, Spain

**Keywords:** Diet, Nutrient intake, Food frequency questionnaire, Pregnancy, Validity

## Abstract

**Background:**

Studies exploring the role of diet during pregnancy are still scarce, in part due to the complexity of measuring diet and to the lack of valid instruments. The aim of this study was to examine the reproducibility and validity (against biochemical biomarkers) of a semi-quantitative food frequency questionnaire (FFQ) in pregnant women.

**Methods:**

Participants were 740 pregnant women from a population-based birth cohort study in Valencia (INMA Study). We compared nutrient and food intakes from FFQs estimated for two periods of pregnancy (reproducibility), and compared energy-adjusted intake of several carotenoids, folate, vitamin B12, vitamin C and α-tocopherol of the FFQ in the first trimester with their concentration in blood specimens (validity).

**Results:**

Significant correlations for reproducibility were found for major food groups and nutrients but not for lycopene (r=0.06); the average correlation coefficients for daily intake were 0.51 for food groups and 0.61 for nutrients. For validity, statistically significant correlations were observed for vitamin C (0.18), α-carotene (0.32), β-carotene (0.22), lutein-zeaxantin (0.29) and β-cryptoxantin(0.26); non-significant correlations were observed for retinol, lycopene, α-tocopherol, vitamin B12 and folate (r≤0.12). When dietary supplement use was considered, correlations were substantially improved for folate (0.53) and to a lesser extent for vitamin B12 (0.12) and vitamin C (0.20).

**Conclusion:**

This study supports that the FFQ has a good reproducibility for nutrient and food intake, and can provide a valid estimate of several important nutrients during pregnancy.

## Introduction

Nutrition during pregnancy plays an important role in the well-being of the mother and fetus, and may further influence the health of the children later in life [1,2]. The ability to assess the role of a complex exposure such as maternal diet during pregnancy requires valid instruments.

Food records and 24-h dietary recalls may provide accurate information on diet although they are expensive to administer and analyze in epidemiological studies. Furthermore, food records require a high level of cooperation and literacy and several days would be required to evaluate the long-term intake of foods and nutrients which makes them less feasible [3]. At present, food frequency questionnaires (FFQ) are the preferred dietary assessment method in most epidemiological studies mainly due to their low cost and ease of administration and, therefore, they have been validated in many different populations [3,4]. However, FFQ have been less often validated to assess diet during pregnancy, a period when many dietary changes occur and the use of dietary supplements is common [5]. The most frequent reference methods to validate FFQ have been food records and 24-h recalls [6] although they have been used less frequently in pregnant women. Thus, biomarkers for nutrient intake may be an alternative reference method for the validation of some nutrient intakes since their measurement errors are independent from those of FFQ [7,8].

In this study, as part of the on-going research of the INMA Project, the aim of which was to investigate the role of environmental pollutants in air, water and diet during pregnancy and early childhood in relation to child growth and development [9,10] we evaluated the reproducibility of a semi-quantitative FFQ for assessing usual dietary intake during pregnancy; we also examined the validity for the assessment of the usual intake of several nutrients during early pregnancy by comparing dietary intakes estimated by the FFQ with blood levels of these nutrients.

## Methods

### Study population

Participants were 740 healthy pregnant women from a mother and child prospective cohort study in Valencia, one of the cohorts of the INMA Project started in Spain in 2003 [9,10]. Details of the recruitment and follow-up have been described previously [11,12]. Briefly, pregnant women from a well-defined geographic area, attending the first prenatal visit at the hospital, were recruited before week 13 of gestation. Of the 840 women initially enrolled in the study between February 2004 and June 2005, 787 (93.7%) gave birth to a singleton live infant between May 2004 and February 2006. The final analysis was based on 740 (88.0%) women who completed the FFQ at weeks 12 (weeks 10–13 of gestation) and 32 of pregnancy (weeks 28–32 of gestation), and provided blood samples for the validation study. All subjects gave informed consent. The study protocol was approved by the Hospital Ethics Committee.

### Covariates

Information on sociodemographic factors, parental anthropometric measures, maternal smoking, and newborn-related variables was obtained from a questionnaire administered in the first and third trimesters of pregnancy (approximately weeks 12 and 32) and from maternal and neonatal medical records. The socioeconomic status of pregnant women was defined in 3 occupational categories according to the Spanish adaptation of the British classification system. Social class I included managerial and senior technical staff and freelance professionals; class II included intermediate occupations and managers in commerce; class III included skilled nonmanual workers; class IV included skilled and partly skilled manual workers; and class V included unskilled manual workers. We used 3 categories (I+II, upper; III, middle; IV+V, lower) in our analysis [13]. Pre-pregnancy body mass index (BMI) was calculated by dividing the self-reported weight before pregnancy in kg by the square of the height in meters. Gestational weight gain was defined and categorized according to the Institute of Medicine guidelines (a weight gain of 12.5–18 kg was low for a pre-pregnancy BMI<19.8, a gain of 11.5–16 kg was normal for a BMI of 19.8–26.0, and a gain of 7–11.5 was high for a BMI> 26.0–29.0) [14]. Smoking in pregnancy was defined as never, first trimester only and entire pregnancy. Alcohol (g/d) intake was assessed in the FFQ with specific items for wine, beer, and liquors, together with standard servings for all the items. We collected detailed information on supplement use by including questions to identify the date of first use, time using it, frequency (times/week), dose and brand names of supplements which allowed us to estimate the daily nutrient intake of supplements for each period of pregnancy.

### Dietary assessment: semiquantitative food frequency questionnaire

We used a semi-quantitative FFQ of 101 food items to assess the usual daily intake of foods and nutrients (available at: http://bibliodieta.umh.es/files/2011/07/CFA101.pdf). The FFQ was a modified version from a previous FFQ based on the Harvard questionnaire [15], which we developed and validated using four 1-week dietary records in an adult population in Valencia. The validity correlation coefficients (adjusted for energy intake) ranged from 0.27 for folate intake to 0.67 for calcium intake (average 0.47), and the reproducibility correlation coefficient s ranged from 0.30 for carotene intake to 0.65 for calcium intake (average 0.40) [16,17]; this is a similar range to other established diet questionnaires [3,4]. For the dietary assessment of pregnant women in the INMA cohort study, we added additional food items in the FFQ in order to capture the major sources of the most relevant nutrients, including specific carotenoids.

Participants in the study were asked twice during pregnancy how often, on average, they had consumed each food item over two periods of several months. The first period covered the time from the last menstruation to the first prenatal visit that occurred between the 10–13 weeks of pregnancy; the second period was the time between the first visit and the second one between weeks 28–32 of gestation. Serving sizes were specified for each food item in the FFQ. The questionnaire had nine possible responses, ranging from ‘never or less than once per month’ to ‘six or more per day’. Additionally, we asked whether study participants followed special diets.

Nutrient values were primarily obtained from the food composition tables of the US Department of Agriculture publications as well as other published sources for Spanish foods and portion sizes [18,19]. In order to obtain average daily nutrient intakes from diet for each individual, we multiplied the frequency of use for each food by the nutrient composition of the portion/serving size specified on the FFQ and added the results across all foods. For those nutrients often used in supplements during pregnancy such as folate, vitamin C and vitamin B12, the total daily nutrient intake was estimated by adding the average daily intake from supplements and the usual daily nutrient intake from the FFQ. In order to convert folic acid intake from supplements to dietary folate, we used the equivalence of 1 mcg of folate in the diet equals to 0.6 mcg of folic acid from supplements [20]. We estimated the mean daily consumption for 17 foods and food groups by grouping the intake of specific foods in the FFQ (Table 1).

**Table 1:** Definition of food groups

**Food groups**	**Foods**
Dairy Products (11)	whole milk; semi-skimmed milk; skimmed or low fat milk; condensed milk; full cream; whole and low fat yogurt; whole and low-fat cheese; custard, cream caramel, pudding; ice-cream
white meat (3)	Chicken or turkey with; and without skin; game (duck, quail, rabbit)
Red meat (4)	Beef, pork or lamb; liver; offal; hamburger
Processed meat (4)	ham, salami and others; sausages; pate; bacon
Lean fish (2)	hake, sole, gilthead and similar white fish type; assorted or mixed fried fish
Fatty fish (5)	swordfish, bonito, and fresh tuna; small oily fish (mackerel, sardine; anchovy); canned tuna; canned sardine or mackerel; dry or smoked fish.
Seafood (4)	clams, mussels; squid, octopus; shellfish (cramps, shrimps, lobster); surimi and other fish-based food products
Fruits (10)	oranges; orange juice; bananas; apples or pears; peaches, nectarines, or apricots; watermelon or melon; grapes; prunes or plums; kiwis; olives
Vegetables (12)	spinach; cabbage, cauliflower or broccoli; lettuce or endive; tomatoes; onions; carrots or squash; green beans; eggplant, zucchini, or cucumber; green, red, or yellow peppers; artichokes; asparagus; and garlic
Cereals and Pasta (4)	Breakfast cereals; corn; rice; pasta
Bread (2)	white and whole breads
Sweets and sugar (7)	biscuits; cookies; baked goods; added sugar; marmalade, honey; chocolate; chocolate/cocoa powder
Vegetable Fat (4)	Olive oil; sunflower, corn oils; margarine; mayonnaise

### Biomarkers

Nonfasting blood samples were obtained from each participant during the first visit between weeks 10–13th of pregnancy and analysed in a central laboratory (Queen’s University, Belfast). All participants were instructed by the interviewers not to consume fruits, vegetables or juice for breakfast on the day of the blood test. A thorough protocol was designed to collect, transport and measure the blood samples for vitamin C, E, B12, folate and carotenoids. Blood samples were separated by centrifugation and stored at -80°C. The blood samples for vitamin C determination were collected at clinical examination under subdued light, wrapped in tin foil, stabilized with meta-phosphoric acid and placed in insulated dry containers at 4°C to exclude light and, therefore, avoid vitamin C degradation. Blood samples packed in dry ice were shipped to the central laboratory by dedicated couriers. Plasma cholesterol was measured to adjust carotenoid concentrations. Folate and vitamin B12 concentrations in serum were measured using a commercially available radioassay (SimulTRAC-SNB ICN Pharmaceuticals, California, USA). Serum carotenoids were measured by HPLC with diode array detection as described by Craft [21]. Lutein+zeaxanthin plasma concentrations were combined as information for these nutrients is combined in the main food composition tables. Serum concentrations of α-tocopherol were measured by high-performance liquid chromatography (HPLC) with UV detection at 292 nm [21]. Plasma vitamin C was measured using an ascorbate oxidase-based assay as described by Vuillemier & Keck [22]. The inter-assay CV were <10.0% and intra-assay CV<5.0% for all species. The assays were standardized against the appropriate National Institute of Standards and Technology standard reference materials.

### Statistical analysis

Data analyses were performed with the STATA statistical software package (Stata® release 9, 2005). We calculated means and standard deviations for total nutrient intakes and food consumption from the FFQ, and for biomarkers. We used paired Student’s test for means comparison of the individual daily nutrient intakes and food consumption reported in the two periods.

All nutrient and food group intakes were log-transformed prior to analysis to improve their normality. Energy-adjusted intakes were computed using the residual method, where each nutrient is regressed on total calories, and the population mean was then added back to the calculated residuals [15]. Since most carotenoids are transported in plasma lipoproteins, plasma concentrations of carotenoids and vitamin E were also adjusted per plasma cholesterol concentrations using the residual method.

To assess the reproducibility of the FFQ, we estimated Pearson correlations to compare the individual energy-adjusted dietary intakes of nutrients and foods reported from the first and second interviews. Pearson correlations were also used to evaluate the validity of the FFQ by comparing individual energy-adjusted dietary intakes from the first FFQ and their respective plasma concentrations of the nutrients vitamin C, E, B12, folate and carotenoids (α- carotene, β- carotene, lutein + zeaxanthin, lycopene and β-cryptoxanthin). Spearman correlation coefficients were also estimated although the results were very similar to those observed for parametric correlations. Therefore, only Pearson correlations are presented. Correlation coefficients were also estimated taking into consideration supplement use and according other variables such as season of the year.

## Results

Table 2 presents the main characteristics of the 740 pregnant women: 45.4% of women were under 30 years of age, 88.0% were born in Spain, 28.9% were overweight or obese before pregnancy, and the mean gestational age was 39.5 weeks. A large proportion of women (23%) smoked during all pregnancy. Supplement use of folic acid and vitamins C and B12 during pregnancy was reported by 94.9%, 55.8% and 87.2% of women respectively.

**Table 2:** Characteristics of participant pregnant women of the Inma–Valencia study, 2004–2006

**Characteristics**	**Subjects (%)**
Maternal age (in years)	
≤ 29	336 (45)
30–34	285 (39)
≥35	119 (16)
Educational level	
≤Primary School	249 (34)
Secondary School	314 (42)
University	177 (24)
Country of origin	
Spain	651 (88)
Other countries	89 (12)
Socioeconomic status categories	
I+II, upper	118 (16)
III, middle	178 (24)
IV+V, lower	444 (60)
Pre-pregnancy body mass index (in kg/m^2^)	
<25	526 (71)
25.0–29.9	139 (19)
≥ 30	75 (10)
Smoking during pregnancy	
No	439 (59)
First trimester only	128 (17)
All pregnancy	173 (24)
Alcohol intake	
None	518 (70)
0–1 g/d	146 (20)
>1 g/d	76 (10)
Gestational weight gain *(missing=13)*	
Normal	261 (36)
Low	179 (25)
High	287 (39)
Parity	
0	407 (55)
≥1	333 (45)
Medical problems in previous pregnancies *(missing n=6)*	
Yes	150 (20)
Use of supplement containing (any time in pregnancy):	
Folic acid *(missing=32)*	675 (95)
Vitamin C	413 (56)
Vitamin B12	645 (87)

(N=740).

### Reproducibility

Mean daily intakes of nutrients and food groups based on the FFQs are presented in Table 3. In general, intakes of energy and most of nutrients were slightly lower in the second period of pregnancy (*P*<0.05), however some carotenoids (eg., lycopene 13.5% more), vitamin D and calcium showed higher intakes in the second period. The Pearson correlation coefficients for nutrients estimated by the two FFQ are also presented in Table 3. Highly significant correlations were observed for most nutrients, ranging from r=0.14 for lycopene to r=0.70 for iodine. The average of correlation coefficients was 0.51. When the analysis was based on energy adjusted nutrient intakes, the magnitude of correlation coefficients was reduced for most nutrients although they were all statistically significant except for lycopene.

**Table 3:** Mean daily nutrient and food intakes and Pearson correlation coefficients among pregnant women of the INMA-Valencia Study, 2004–2006

	**FFQ1**^**a**^	**FFQ2 **^**a**^	***P ***^**b**^	**Pearson coefficient correlations between FFQ1 and FFQ2**		**Percent of agreement **^**e**^
	**Mean (SD)**	**Mean (SD)**			**Unadjusted **^**c**^	**Adjusted **^**d**^	
**Nutrients** (units/day)						
Energy (kcals)	2304 (587)	2212 (633)	<0.001		0.58		75.4
Protein (g)	102 (25)	99 (27)	<0.001		0.53	0.49	75.8
Total carbohydrates (g)	261 (81)	253 (86)	0.005		0.57	0.36	73.8
Dietary fiber (g)	24 (8)	22 (8)	<0.001		0.48	0.43	68.8
Cholesterol (mg)	340 (109)	332 (113)	0.070		0.48	0.35	72.0
Total fat (g)	99 (29)	93 (31)	<0.001		0.50	0.35	74.2
SFA (g/)	31 (11)	30 (11)	0.026		0.51	0.38	72.7
MUFA (g)	46 (14)	43 (14)	<0.001		0.46	0.35	69.6
PUFA (g)	15 (6)	14 (7)	0.001		0.49	0.35	73.0
Omega 3 (g)	1.6 (0.5)	1.5 (0.5)	<0.001		0.53	0.50	71.9
Omega 6 (g)	13 (6)	12 (6)	0.001		0.49	0.35	73.0
Retinol (μg)	812 (819)	844 (781)	0.335		0.51	0.53	76.2
α- carotene (μg)	536 (468)	532 (525)	0.859		0.50	0.51	71.6
β- carotene (μg)	4499 (2438)	4553 (2665)	0.583		0.55	0.56	72.7
Lutein+Zeaxanthin (μg)	3157 (2455)	3091 (2381)	0.453		0.59	0.59	75.7
Lycopene (μg)	4410 (2727)	5004 (3763)	<0.001		0.14	0.06*	57.7
β- Cryptoxanthin (μg)	360 (253)	362 (263)	0.851		0.38	0.36	67.0
Vitamin B6 (mg)	2.1 (0.7)	2.1 (0.8)	0.684		0.53	0.50	73.4
Folate (μg)	305 (99)	297 (106)	0.034		0.52	0.48	71.5
Vitamin B12 (μg)	9.9 (5.7)	9.7 (5.5)	0.335		0.46	0.43	70.8
Vitamin C (mg)	144 (83)	143 (86)	0.720		0.45	0.44	71.7
Vitamin D (μg)	3.1 (1.9)	3.3 (2.1))	0.009		0.55	0.52	71.5
Vitamin E (mg)	11.4. (4.1)	10.8 (4.5)	0.001		0.45	0.37	70.1
Calcium (mg)	1289 (429)	1320 (455)	0.060		0.51	0.49	71.4
Iron (mg)	21 (6)	20 (6)	<0.001		0.53	0.40	75.0
Magnesium (mg)	387 (105)	383 (107)	0.215		0.69	0.57	72.7
Sodium(mg)	3411 (996)	3171 (1043)	<0.001		0.49	0.33	72.8
Zinc (mg)	28 (7)	26 (7)	<0.001		0.52	0.40	74.1
Iodine ^f^ (μg)	222 (88)	226 (84)	0.151		0.70	0.72	80.5
**Food groups (g/day)**							
Dairy Products	468 (245)	520 (262)	<0.001		0.55	0.44	72.8
Eggs	20 (10)	21 (11)	0.288		0.39	0.36	96.2
White meat	33 (18)	32 (23)	0.129		0.38	0.37	78.4
Red meat	60 (32)	54 (29)	<0.001		0.40	0.36	68.4
Processed meat	42 (33)	39 (33)	0.034		0.36	0.33	66.8
Lean fish	25 (20)	25 (20)	0.264		0.36	0.36	70.9
Fatty fish	28 (23)	28 (24)	0.857		0.41	0.41	68.6
Seafood	11 (11)	10 (9)	<0.001		0.43	0.40	70.8
Fruits	293 (204)	320 (217)	0.004		0.41	0.38	59.3
Vegetables	216 (121)	213 (126)	0.554		0.61	0.62	59.5
Nuts	6 (11)	5 (7)	0.003		0.23	0.17	79.1
Legumes	31 (25)	28 (23)	0.005		0.39	0.38	93.0
Cereals and Pasta	119 (51)	114 (49)	0.015		0.48	0.43	71.5
Bread	103 (72)	86 (65)	<0.001		0.38	0.35	64.3
Potatoes	60 (38)	55 (37)	0.001		0.39	0.33	66.9
Sweets and sugar	53 (44)	54 (48)	0.438		0.44	0.37	69.5
Vegetable fat	24 (14)	22 (14)	<0.001		0.30	0.30	64.3

^a^ FFQ1 administered between weeks 10–13^th^ and FFQ2 between weeks 28–32^nd^ of gestation; ^b^ P-value from paired *t*-test; ^c^ Nutrient crude intakes were log-transformed; ^d^ Adjusted for total energy intake; ^e^ Overall percentage categorized in the same or an adjacent quintile; ^f^ Iodine intake from diet and use of iodized salt; ^*^P>0.05, all other coefficients, *P*<0.01; SFA, Saturated fatty acids; MUFA, monounsaturated fatty acids; PUFA, Polyunsaturated fatty acids.

(n=740).

Regarding food group intake, the mean intake for eight food groups estimated by the second FFQ was significantly lower than by the first FFQ (red meat, processed meat, seafood, nuts, legumes, cereals and pasta, bread, potatoes and vegetable fat) whereas the consumption of dairy products and fruits was significantly higher (Table 3). The correlation coefficients of food groups between the two FFQ were in general lower than those observed for nutrients, ranging from r=0.17 for nuts to r=0.61 for vegetables. The average correlation of absolute food group intakes between the two FFQ was r=0.39. As for nutrients, the energy-adjusted correlations for food groups were slightly lower than unadjusted correlations although all of them were statistically significant (except for nuts).

According to classification into quintiles of nutrient intakes as estimated by the two FFQ, between 57.7% (lycopene) and 80.5% (iodine) of women were classified in the same or adjacent quintile (Table 2). Regarding the intake of food groups, between 59.3% (fruits) and 96.2% (eggs) of women were in good agreement, i.e., classified in the same or adjacent quintiles by both FFQ (energy adjusted intakes.

### Validity

Table 4 shows the mean daily intake for ten nutrients and fruits and vegetables, and the correlation coefficients between the first FFQ and plasma concentration (relative validity). The correlation coefficients based on energy-adjusted nutrient intakes and cholesterol-adjusted plasma concentrations were slightly higher than the unadjusted data. The lowest coefficients were observed for lycopene (r=0.04), retinol (r= 0.05) and vitamin E (r=0.05) and the highest for β-carotene (r=0.32). The average correlation coefficient for energy-adjusted nutrients was r=0.16. When correlation analyses were performed for total nutrient intakes of folic acid, vitamin B12 and C (i.e., based on the intake from diet and supplements), correlation coefficients improved substantially, particularly for folate, from 0.12 to 0.53 (Table 3). The correlation coefficient between fruit and vegetable intake and plasma concentration of carotenoids (lycopene not included) was r=0.28.

**Table 4:** **Mean daily nutrient and food intakes by the ffq at week 12**^**th **^**and nutrient plasma concentrations and pearson correlation coefficients in pregnant women of the Inma-Valencia study, 2004–2006**

**Nutrients and foods**	**FFQ1**	**Plasma concentration**	**Pearson coefficient correlations between FFQ1 and plasma concentrations**		**Agreement (%) **^**c**^
	**Mean**	**Mean (SD)**	***r ***^***a***^			***r ***^***b ***^**adj.**
Retinol (μmol/l)	812	1.78 (0.54)	0.02		0.05	52.3
α- carotene (μmol/l)	536	0.15 (0.14)	0.31**		0.32**	61.9
β- carotene (μmol/l)	4499	0.40 (0.30)	0.21**		0.22**	58.8
Lutein + Zeaxanthin (μmol/l)	3157	0.32 (0.13)	0.26**		0.29**	60.1
Lycopene (μmol/l)	4410	0.72 (0.62)	0.05		0.04	52.3
β-cryptoxanthin (μmol/l)	360	0.22 (0.15)	0.26**		0.26**	62.3
Folate (mmol/dl)	305	80 (211)	0.06		0.12**	51.1
Diet + supplements (n=708) ^d^	1616		0.53**		0.53**	72.0
Vitamin B12 (pmol/l)	9.9	322 (121)	0.08*		0.08*	55.3
Diet + supplements (n=740) ^d^	12.0		0.11**		0.12**	55.0
Vitamin C (μmol/l)	144	50 (22)	0.16**		0.18**	57.4
Diet + supplements (n=520) ^d^	157		0.18**		0.20**	58.3
Vitamin E (μmol/l)	11.4	33.2 (7.4)	0.01		0.05	52.6
Fruits and vegetables (g/day), mean (SD) *vs*	508 (260)		0.25**	0.28**	61.7	
Carotenoids (μmol/l)^e^		1.09 (0.56)				

^a^ Nutrient intakes were log-transformed; ^b^ Adjusted for total energy intake and plasma carotenoids and vitamin E adjusted for total cholesterol; ^c^ Overall proportion categorized in the same or an adjacent quintile based on energy-adjusted intakes; ^d^ Total nutrient intake from diet and supplements. Folic acid from supplements were converted to dietary folate (0.6 μg from supplements =1 μg from diet); plasma concentration of vitamin C was available for 520 women; ^e^ Carotenoid in plasma included α-carotene, β-carotene, Lutein+Zeaxanthin, β- Cryptoxanthin; ^*^P <0.05; ^**^P<0.01.

We explored if validity increased by comparing nutrient plasma concentrations with the average of the two administrations of the FFQ at weeks 12 and 32. Correlations substantially improved for lycopene (from r=0.04 to r=0.20) and slightly for α-carotene, β-carotene and β-cryptoxantin, but not so for others the nutrients. We also explored if the season of the year had influence on correlations. Lycopene correlations for samples taken in spring (n=259) and summer (n=208) were significantly higher than the other two seasons or the overall, r=0.22 and r=0.23 respectively. For retinol, we also observed a better correlation in winter (n=130, r=0.26).

## Discussion

The results of this study indicate that the FFQ is a reasonably good method for dietary assessment among pregnant women in Spain. The FFQ showed a good degree of reproducibility for most of the foods and nutrient intakes, and an acceptable relative validity for some nutrients of special interest during pregnancy. This validation study was undertaken because a new FFQ has been developed for assessment of the dietary intakes of pregnant women in the INMA-Project, a prospective cohort study of pregnant women and their offspring [9,10]. On the other hand, this is the first study ever done to validate a FFQ among pregnant women in Spain with a sample size large enough to detect as statistically significant even small correlation coefficients which may be considered as minimally valid (r>0.20).

The correlation coefficients for most of the nutrients and food groups were comparable with those observed in other validation studies of FFQ among pregnant women and other populations [3,5]. Reproducibility was assessed by comparing the results from the FFQ at two different periods in pregnancy while validity was explored by comparing the nutrient intakes from FFQ and biomarker concentrations of several nutrients in plasma that are potentially important for perinatal research and that are sensitive to dietary intake (carotenoids, folate, vitamin B12 and C, E and retinol). Despite the use of biomarkers as a reference methods may have some limitations since they do not provide a quantitative measure of dietary intake but rather a qualitative indicator and may be more related to recent intake while FFQ to long-term exposure, plasma concentration for some nutrients are sensitive and acceptable indicators of intakes. Therefore, their use for FFQ validation is considered appropriate since the two methods of assessing diet have different sources of error that are unlikely to be correlated with each other [7,8].

Overall, our FFQ has shown good reproducibility and therefore, it may be considered a reliable dietary assessment method among pregnant women of the INMA Project in Spain. Few studies have been published presenting data on the reproducibility of their FFQs in pregnant women [23-27]. In our study, the average of correlation coefficients between the first and second administration of the FFQ was 0.51 for the intakes of the 29 nutrients considered. Similar results were observed in a study with low-income pregnant women, except for a stronger vitamin A correlation [27]. In a Finnish study, the average of correlation coefficients for all nutrients considered was 0.66, higher than the 0.51 in our study [26]. In a Portuguese study, the estimated average of coefficients for 15 nutrients was also high (0.62), although the interval of the FFQ administration in this study was short, two weeks in the third trimester of pregnancy [24]. In a recently published study with pregnant women in Malaysia [23], the average of correlation coefficients for nutrients and foods was very high 0.87, which may be related in part to the short time period of administration between FFQ1 and FFQ2 (10 weeks) and to a more stable diet in the third trimester of pregnancy in that country. We applied a 20-week interval which made answer memorization more unlikely, thus avoiding an artificially increased reproducibility. However, a long interval could also be a concern since diet in pregnant women may not be as stable as among non-pregnant women and reproducibility may be compromised by real dietary changes. Although some studies have reported higher nutrient intakes with the second FFQ administration [26,27], other studies have shown no evidence of major changes in diet during pregnancy [24]. We observed slightly higher intakes for many foods and nutrients during the first period (but not for lycopene, fruits and dairy products), which may support the idea that some changes in diet did occur. Therefore, we explored whether reproducibility correlations were modified by categories of variables such as self-reported changes in diet (no/yes), age, body mass index, country of origin and self-reported vomiting during pregnancy (no/yes); however, correlations remained practically unchanged (data not shown). Despite potential changes in diet during pregnancy, the FFQ showed a satisfactory level of reproducibility for most foods and nutrients, particularly for those more frequently eaten. As far as we know, only two studies have previously explored reproducibility of food groups by FFQ among pregnant women [23,26]. Although foods were not grouped in the same way in our study, the average of the correlation coefficients were very similar to those observed in the Finnish Study while slightly lower than those reported in other non-pregnant populations [5].

Regarding the relative validity of the FFQ as assessed by biomarkers, poor correlations were found between dietary intake and plasma concentrations of retinol and α-tocopherol (r=0.05). A low validity has been also found in other studies with pregnant women [28-31] as well as non-pregnant population [4,32]. The retinol concentrations in plasma is highly regulated by liver stores over a wide range of dietary intakes that can be found mainly in subjects with either severely depleted or highly saturated liver stores [32]. Concerning the poor correlation for vitamin E, it has been suggested that plasma concentration may not be a good marker for usual nutrient intake among pregnant women and that other tissues (e.g., adipose tissue) may better represent usual vitamin E intake [3]. Nevertheless the lack of validity for retinol and vitamin E deserves attention when using this FFQ in the study of diet disease relationships.

The correlation coefficient for lycopene was lower than the observed for other carotene. It has been suggested that in order to increase validity for the assessment of long-term intake, one approach would be to use the average of repeated administrations of a dietary questionnaire [33]. Correlations for lycopene were substantially improved when plasma concentration was compared with the average of the two administrations of the FFQ. Correlation coefficients for α-carotene, β-carotene and β-cryptoxantin were slightly improved when we used the average of the two FFQ, but not so for the others nutrients (data not shown). Poor correlations could be also related to when blood samples and FFQ were collected since the availability of foods with high content of carotenoids may differ substantially according to the season of the year. When the analysis was performed according to the season of the year, lycopene correlations for samples taken in spring and summer were significantly higher than the other two seasons or the overall. For retinol, we observed a better correlation in winter. Thus, validity of the FFQ may be even higher than shown in Table 4 as correlations were improved when time frame and season between nutrient intakes and plasma concentrations were matched more properly in the analysis.

Despite the fact that some correlations were low, our results support that the FFQ may be valid to estimate of long-term intakes and acceptably classify women according to their intakes for relevant nutrients. In fact, correlations in this study were slightly higher than those found in a previous study we performed using a similar FFQ in an elderly population [17] and in agreement with other studies in pregnant as well as non-pregnant populations [3-5].

On the other hand, the correlation coefficients were substantially improved for folate (from r=0.12 to r=0.53) and to lesser extent for vitamin B12 (from r=0.08 to r=0.12) and vitamin C (from r=0.18 to r=0.20) when dietary supplement use was considered. In Spain, the contribution of vitamin B12 from supplements to the total intake of this vitamin is probably low although it may be included in some folic acid and multivitamin supplements, and therefore may influence the total dietary intake of this vitamin in some periods of pregnancy [34,35].

Other methods of dietary assessment such as food diaries or 24 h recalls are commonly used as a reference in validation studies of FFQ [6], and this may be a limitation of this study. However, the use of these methods was problematic and unfeasible in our study because of the large sample size and the considerable proportion of women with low educational level and with a country of origin different from Spain.

Therefore, we only explored relative validity by comparing nutrient intakes with their respective concentrations in plasma. Nutrient levels in plasma may be influenced not only by dietary intake but also by external factors such as the food matrix, the food preparation and by host factors such as gender, smoking status and BMI; however, when we stratified the analysis for some of these variables, correlations did not change and showed acceptable degree of validity still.

In conclusion, our findings show that reproducibility and validity of the FFQ assessed in this study using biological markers were acceptable and comparable with the results of earlier studies. We conclude that our FFQ is a good method for assessing intake of several relevant nutrients during pregnancy.

## Competing interests

The authors declare that there are no conflicts of interest.

## Authors’ contributions

All the authors contributed to the preparation of the paper and read and approved the final manuscript. J.V. wrote the manuscript and designed the study. J.V., E.M. N-M., D. G-M., M. G-H., F.G., I.S.Y., R.R, F.B., M.M., M.R., C.I., made substantial contributions to acquisition of data, analysis and interpretation of data, and drafting and revising critically the manuscript. I.S.Y contributed to nutrient determinations in blood samples. All authors reviewed the final version of the manuscript.

## Authors’ informations

*Members of the INMA Valencia Cohort Study*: Jesús Vioque, Eva-María Navarrete-Muñoz, Daniel Gimenez-Monzó, Manuela García de la Hera, Sandra González Palacios, Marisa Rebagliato, Ferran Ballester, Mario Murcia, Carmen Iñiguez, Rosa Ramón, Rosa Ramón, Ana Esplugues, Clara Rodriguez-Bernal, Alfredo Marco, Sabrina Llop, Marisa Estarlich, Amparo Cases.
